# Abdominal DIBH reduces the cardiac dose even further: a prospective analysis

**DOI:** 10.1186/s13014-018-1062-6

**Published:** 2018-06-22

**Authors:** Feng Zhao, Jiayan Shen, Zhongjie Lu, Yongbiao Luo, Guorong Yao, Luyi Bu, Jia Ge, Xin Yang, Lihua Ning, Senxiang Yan

**Affiliations:** 1 Department of Radiation Oncology, the First Affiliated Hospital, College of Medicine, Zhejiang University, Hangzhou, Zhejiang 310003 People’s Republic of China; 2 Department of Radiation Oncology, Yiwu Central Hospital, Yiwu, Zhejiang, 322000 People’s Republic of China

**Keywords:** Breast cancer, Breath holding, 3-dimensional conformal radiation therapy (3D-CRT), Intensity-modulated radiation therapy (IMRT), Organs at risk, Radiotherapy dosage

## Abstract

**Background:**

Deep inspiration breath hold (DIBH) can be performed using different breathing maneuvers, such as DIBH with a thoracic breathing maneuver (T-DIBH) and DIBH with an abdominal breathing maneuver (A-DIBH). Dosimetric benefits of A-DIBH were investigated in the treatment of left-sided breast cancer radiotherapy (RT) with both 3-Dimensional conformal radiation therapy (3D-CRT) and intensity-modulated radiotherapy (IMRT) techniques.

**Methods:**

Twenty-two patients with left-sided breast cancer were enrolled in this study. 3D-CRT and IMRT plans were generated for each patient with three different CT scans of free breathing (FB), T-DIBH and A-DIBH. There were total of six treatment plans generated for each patient: FB_3D-CRT; TDIBH_3D-CRT; ADIBH_3D-CRT; FB-IMRT; TDIBH-IMRT; ADIBH-IMRT. Doses to the heart, left anterior descending coronary artery (LADCA), and ipsilateral lung were evaluated and compared using the Wilcoxon signed-rank test.

**Results:**

The mean doses to the heart, LADCA and ipsilateral lung in 3D-CRT plans generated from 3D-CRT with FB, T-DIBH and A-DIBH were (2.89 ± 1.30), (1.67 ± 0.90) and (1.34 ± 0.43) Gy (all *P* < 0.05), respectively, with FB; (29.08 ± 16.72), (13.94 ± 14.74) and (10.22 ± 10.30) Gy (all P < 0.05), respectively, with T-DIBH; and (7.77 ± 2.71), (7.32 ± 1.42) and (6.90 ± 1.60) Gy (all P < 0.05), respectively, with A-DIBH. The mean doses to the heart, LADCA and ipsilateral lung in IMRT plans were generated from IMRT with FB, T-DIBH and A-DIBH were (1.96 ± 2.25), (1.37 ± 0.44) and (1.18 ± 0.26) Gy (all P < 0.05), respectively, with FB; (16.10 ± 7.45), (8.6 ± 6.60) and (7.35 ± 5.42) Gy (all P < 0.05), respectively, with T-DIBH; and (5.90 ± 2.24), (5.65 ± 1.58) and (5.62 ± 1.05) Gy (all *P* > 0.05), respectively, with A-DIBH.

**Conclusions:**

This study indicates that both 3D-CRT and IMRT plans with A-DIBH achieved lower cardiac and LADCA doses than plans with FB and T-DIBH; 3D-CRT plans with A-DIBH achieved lower ipsilateral lung doses than plans with FB and T-DIBH; and IMRT plans with A-DIBH had better outcomes than 3D-CRT plans with A-DIBH with respect to the mean dose to the heart, LADCA and ipsilateral lung. IMRT plans with A-DIBH should be incorporated into the daily routine for left-sided breast RT.

## Background

The standard treatment for patients with early-stage breast cancer is breast-conserving surgery followed by adjuvant radiotherapy (RT) [1]. Adjuvant RT of the whole breast after breast-conserving surgery has been shown to improve local control and overall survival in breast cancer patients [2]. However, heart and lung exposure to radiation may result in late radiation-induced cardiac and pulmonary complications, including heart disease and lung cancer [3]. Of particular note, due to the close proximity of the chest wall to critical anterior cardiac structures, e.g., the left anterior descending coronary artery (LADCA), these structures are frequently included in the irradiation field, so patients undergoing RT for left-sided breast cancer are at increased risk for changes in cardiac perfusion as well as late cardiac morbidity and mortality [4]. Evidence suggests that there is a dose-response relationship between the heart and the radiation dose and that the rate of ischemic heart disease is proportional to the mean dose to the heart [5, 6]. Moreover, Sardaro et al. [7] reported that a 1 Gy increase in the mean heart dose is equal to a 4% increase in the risk of late heart disease. More recently, Darby et al. [8] estimated a relative 7.4% increase in the rate of major coronary events per 1 Gy increase in the mean radiation dose to the heart among patients with breast cancer receiving adjuvant RT from 1958 to 2001.

Consequently, in recent years, a large effort has been made to develop techniques to shield the heart and minimize the heart and LADCA doses. These techniques include deep inspiration breath hold (DIBH), intensity-modulated radiation therapy (IMRT) techniques, treatment in the prone position, and proton therapy. One of the most popular strategies is DIBH, which requires the patient to take and hold a deep breath during CT simulation and RT. The DIBH technique was first reported by Sixel and colleagues in a small cohort of patients in 2001 [9]. Since then, for the past 15 years, the benefits of DIBH for left-sided breast cancer have been studied. As reported [10–18], the DIBH technique increases the spatial separation between the heart and the target volume and decreases the volume of the heart within the irradiated field, resulting in a reduction of the cardiac dose without compromising the target coverage.

DIBH can be performed using different breathing maneuvers, including DIBH with a thoracic breathing maneuver (T-DIBH), which predominately uses the diaphragm and chest muscles during inspiration, and DIBH with an abdominal breathing maneuver (A-DIBH), which predominately uses the abdominal muscles during inspiration. Predominately thoracic DIBH and predominately abdominal DIBH may each induce a difference in the spatial separation between the heart and the target volume. Plathow et al. measured external thorax motion during the breathing cycle by using dynamic magnetic resonance imaging of the thorax and found that with the same spirometric vital capacity, DIBH achieved with thoracic breathing and DIBH achieved with abdominal breathing led to different levels of chest wall expansion, with an average difference of 1.9 cm [19]. To the best of our knowledge, the impact of different breathing maneuvers for DIBH (T-DIBH and A-DIBH) during RT for left-sided breast cancer has not been reported before.

Therefore, this study is the first to report on DIBH using different breathing maneuvers during left-sided whole-breast irradiation after breast-conserving surgery. In the current study, we sought to establish whether different breathing maneuvers for DIBH can induce different dosimetric distributions in the heart, LADCA and ipsilateral lung in 3-dimensional conformal radiation therapy (3D-CRT) and IMRT plans, and to quantify which type of DIBH can achieve more benefits in terms of reducing the doses to normal tissues and organs at risk (OAR) from the dosimetric aspect, without compromising the target coverage.

## Methods

### Patients

This prospective study was approved by the Institutional Ethics Review Board. Informed consent was obtained from all individual participants included in the study. The study was conducted in compliance with the ethical principles of the Declaration of Helsinki. Starting in Feb. 2016, 22 consecutive patients in whom adjuvant RT was administered following breast-conserving surgery for breast carcinoma of the left side were enrolled in our institutional DIBH protocol. The mean age was 46.9 years (range, 29–69 years), and the median age was 48 years.

### Respiratory practicing and monitoring

All patients were trained in and practiced thoracic and abdominal DIBH according to audio and visual coaching for at least 1 week prior to the simulation scan. The following instructions were provided. T-DIBH is a kind of DIBH that use the diaphragm and chest muscles during inspiration, and then hold the breath. The abdomen was relatively motionless during T-DIBH. A-DIBH is another kind of DIBH that use the abdominal muscles during inspiration, and then hold the breath. The thoracic cage was relatively motionless during A-DIBH. The patients needed to repeat each mode to improve reproducibility, and the patients had to be able to hold their breath for 15–20 s.

The Varian Real-time Position Management (RPM) System (Varian Medical Systems, Palo Alto, CA, USA), which is a noninvasive, video-based form of respiratory gating system, was used to facilitate DIBH. For monitoring T-DIBH and A-DIBH, the infrared reflecting marker was placed on the patient’s chest wall (below the xiphoid) or the patient’s abdomen (midway between the umbilicus and rib cage), respectively.

### CT simulation

The patient was placed in the CT simulation position without an immobilization device. Each patient underwent three CT simulation scans with a Siemens Sensation Open 24-slice scanner (Siemens, Forchheim, Germany): the first one with FB, the second one with T-DIBH, and the third one with A-DIBH. All patients were scanned on a supine breast board with arms extended above their head in supports (Varian Medical Systems, Palo Alto, CA, USA). The FB scan was obtained as a reference for treatment setup as well as for comparison purposes in this study.

### Volume delineation

All target volumes and OAR were contoured via three CT series in the Eclipse treatment planning system (Varian Medical Systems, Palo Alto, CA, USA) according to the Danish Breast Cancer Cooperative Group (DBCG) atlas [20] and a heart atlas reported by Feng and colleagues [21]. Contouring of target volumes and OAR was performed by an experienced radiation oncologist (Feng Zhao), and its quality was ensured by a quality assurance review by a second senior radiation oncologist (Senxiang Yan).

The clinical target volume (CTV) of the residual breast included all mammary tissues, as visualized on the CT scan; the contouring was aided by a copper thread placed along the palpated breast tissue prior to the CT scan. An extension of the CTV to the planning target volume (PTV) of 5 mm, limited to 3 mm underneath the skin, was used. OARs were delineated: specifically, the heart, the LADCA, and the ipsilateral lung. The heart was defined by the heart muscle, including the complete pericardia from the lower part of the left pulmonary artery to the apex. The LADCA was contoured with a diameter of 5 mm. The left main coronary artery (LMCA) originates from the left side of the ascending aorta, was also contoured. The ipsilateral lung was contoured and excludes the major airways.

### Planning and dosimetric comparison

All plans were generated by the Eclipse treatment planning system (Varian Medical Systems, Palo Alto, CA, USA). For 3D-CRT with the field-in-field technique as well as for IMRT plans, identical gantry angles and beam energies were used. All treatment plans had to meet the criterion that 97% of the PTV was covered by at least 95% of the isodose (and < 108% of the isodose). The prescribed dose was 50 Gy in 25 fractions to the mean PTV in all cases. Additionally, boost series targeting the tumor bed were planned, consisting of 10–16 Gy in 5–8 fractions. To achieve optimal homogeneity of the data in the present analysis, we compared only the whole-breast irradiation series. For each patient, six virtual treatment plans were generated: FB_3D-CRT, T-DIBH_3D-CRT, A-DIBH_3D-CRT, FB_IMRT, T-DIBH_IMRT, and A-DIBH_IMRT.

Dose-volume histograms (DVHs) were calculated and compared. To investigate the dose homogeneity of PTV, the mean dose (Dmean), maximum dose (D2%), minimum dose (D98%), V105%, and homogeneity index (HI = [(Dmax-Dmin)/50] × 100) were calculated. Furthermore, we compared the dose distributions for the heart, LADCA, and ipsilateral lung using standard defined parameters: volume size; Dmean; D2%; and the percentage of the organ volume receiving at least 5 Gy (V5), 10 Gy (V10), 20 Gy (V20) and 30 Gy (V30).

### Statistics

Statistical evaluation was performed using SPSS 17.0 software (SPSS Inc., Chicago, IL, USA). The two-tailed Wilcoxon signed-rank test was conducted to evaluate the dose metrics for the heart, the LADCA, the ipsilateral lung, and the breast PTV. *P*-values less than 0.05 were considered statistically significant.

## Results

**Fig. 1:**
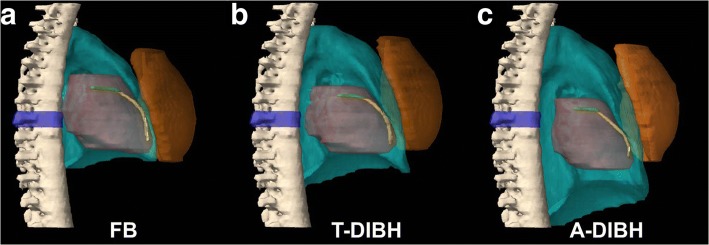
The relative spatial position relationships of the breast CTV (orange), left lung (blue), heart (red), LMCA (green), and LADCA (yellow) in a typical patient during FB (**a**), T-DIBH (**b**) and A-DIBH (**c**). A vertebral body T7 (blue) is contoured as a position marker in the shown case. During T-DIBH, due to expansion of the left lung (B), the heart, LMCA, and LADCA are moved caudally compared with during FB (**a**). During A-DIBH (**c**), the heart, LMCA, and LADCA are moved farther caudally than during T-DIBH (**b**). During T-DIBH (**b**) and A-DIBH (**c**), the breast CTV is moved cranioventrally compared with during FB (**a**) due to the expansion of the left lung

As shown in Fig. 1, exemplary for one patient, during T-DIBH, the heart, LMCA, and LADCA moved caudally compared with those during FB. Of note, the heart, LMCA, and LADCA moved farther caudally during A-DIBH than during T-DIBH. In other words, both DIBH techniques increased the distance between the heart and the breast CTV compared with FB. When A-DIBH and T-DIBH were examined separately, the heart moved farther away from the breast CTV during A-DIBH than during T-DIBH. In addition, the left lung volume increased during T-DIBH and A-DIBH compared with FB.

The volumes of the PTV, heart, LADCA, and left lung with FB, T-DIBH and A-DIBH are shown in Table 1. The differences in PTV and LADCA volumes with FB, T-DIBH, and A-DIBH were not significant. By contrast, the heart volume decreased significantly during T-DIBH and A-DIBH compared with FB. In addition, the left lung volume increased significantly during T-DIBH and A-DIBH compared with FB.

**Table 1:** Volume and dose comparisons among plans generated from 3D-CRT with FB, T-DIBH and A-DIBH

	3D-CRT plans			*P* value		
Parameters	FB	T-DIBH	A-DIBH	FB v.s. T	FB v.s.A	T v.s. A
PTV						
Volume (cm3)	674.14±244.51	673.77±249.54	673.81±246.49	N.S.	N.S.	N.S.
Mean (Gy)	51.61±14.37	51.41±14.65	51.46±13.63	N.S.	N.S.	N.S.
D2% (Gy)	53.88±15.13	53.76±15.37	53.83±13.84	N.S.	N.S.	N.S.
V105% (%)	26.58±22.70	26.51±21.17	26.55±19.27	N.S.	N.S.	N.S.
HI (%)	15.49±1.95	15.34±1.89	15.46±1.72	N.S.	N.S.	N.S.
Heart						
Volume (cm3)	549.54±88.01	504.83±69.55	479.53±74.44	**	***	*
Mean (Gy)	2.89±1.30	1.67±0.90	1.34±0.43	***	***	***
D2% (Gy)	31.25±16.99	12.11±13.22	6.53±4.05	***	***	***
V5 (%)	7.78±4.20	3.68±3.21	2.57±2.05	***	***	**
V10 (%)	4.57±3.07	1.65±2.14	0.93±1.17	***	***	**
V20 (%)	3.26±2.62	1.05±1.68	0.53±0.77	***	***	*
V30 (%)	2.62±2.26	0.78±1.40	0.36±0.57	***	***	*
LADCA						
Volume (cm3)	0.80±0.12	0.80±0.13	0.80±0.10	N.S.	N.S.	N.S.
Mean (Gy)	29.08±16.72	13.94±14.74	10.22±10.30	***	***	***
D2% (Gy)	37.59±17.58	22.03±18.02	18.45±16.52	***	***	**
V5 (%)	87.69±24.39	54.94±39.17	49.42±40.11	***	***	*
V10 (%)	66.02±37.45	32.36±39.20	26.41±37.24	***	***	*
V20 (%)	57.39±41.18	23.37±36.48	18.22±31.57	***	***	*
V30 (%)	52.94±40.24	19.10±34.19	12.55±25.01	***	***	N.S.
Left lung						
Volume (cm3)	1022.22±223.48	1541.81±324.32	1663.36±270.93	***	***	N.S.
Mean (Gy)	7.77±2.71	7.32±1.42	6.90±1.60	*	*	*
D2% (Gy)	46.67±5.06	46.16±2.89	45.31±3.01	*	**	***
V5 (%)	25.53±7.35	25.84±4.13	24.94±4.80	N.S.	N.S.	N.S.
V10 (%)	17.63±6.23	16.99±3.36	15.96±4.05	N.S.	N.S.	*
V20 (%)	13.37±5.77	12.32±3.09	11.26±3.68	N.S.	*	*
V30 (%)	11.34±5.62	10.29±3.15	9.28±3.48	N.S.	*	**

Note: * <0.05, ** <0.01,*** <0.001, N.S.: not significant

Dose comparisons among 3D-CRT plans with FB, T-DIBH and A-DIBH are shown in Table 1. The A-DIBH_3D-CRT plans demonstrated significant reductions in heart Dmean, D2%, V5, V10, V20, and V30 of 19.7, 46.08, 30.16, 43.64, 49.53 and 53.85%, respectively, compared with those in T-DIBH _3D-CRT plans (*P* < 0.05). The A-DIBH_3D-CRT plan displayed further reductions in LADCA Dmean, D2%, V5, V10, and V20 compared with the T-DIBH_3D-CRT plan, with relative reductions of 26.69, 16.25, 10.05, 18.39 and 22.04%, respectively (P < 0.05). For the left lung, the A-DIBH_3D-CRT plan displayed further reductions in Dmean, D2%, V10, V20 and V30 compared with the T-DIBH_3D-CRT plan, with relative reductions of 5.74, 1.84, 6.06, 8.58 and 9.82%, respectively (*P* < 0.05).

Dose comparisons among IMRT plans with FB, T-DIBH and A-DIBH are shown in Table 2. In the IMRT plans, the tendency was similar to that of the 3D-CRT plans for the heart and LADCA, demonstrating that A-DIBH attained the best dosimetric benefit. For the left lung, no significant differences were observed in Dmean among plans with FB, T-DIBH and A-DIBH (*P* > 0.05). Significant differences were observed in only V10 and V20 between A-DIBH and T-DIBH, with relative reductions of 5.91 and 7.87%, respectively, in the A-DIBH_IMRT plan (*P* < 0.05).

**Table 2:** Dose comparisons among plans generated from IMRT with FB, T-DIBH and A-DIBH

	IMRT plans			*P* value		
Parameters	FB_IMRT	T-DIBH_IMRT	A-DIBH_IMRT	FB vs. T	FB vs. A	T vs. A
PTV						
Mean (Gy)	49.43±0.33	49.37±0.47	49.33±0.49	N.S.	N.S.	N.S.
D2% (Gy)	51.46±0.61	51.29±0.61	51.16±0.57	N.S.	N.S.	N.S.
V105% (%)	0.37±0.56	0.36±0.97	0.14±0.43	N.S.	N.S.	N.S.
HI (%)	10.26±1.69	9.83±1.53	9.32±1.34	N.S.	N.S.	N.S.
Heart						
Mean (Gy)	1.96±2.25	1.37±0.44	1.18±0.26	***	***	***
D2% (Gy)	14.67±5.29	7.62±5.29	5.61±3.03	***	***	***
V5 (%)	6.58±3.25	2.83±2.54	1.80±1.46	***	***	***
V10 (%)	4.01±2.32	1.32±1.60	0.68±0.84	***	***	***
V20 (%)	0.59±0.49	0.16±0.29	0.05±0.11	***	***	***
V30 (%)	0.07±0.09	0.01±0.04	0.00±0.00	**	**	N.S.
LADCA						
Mean (Gy)	16.10±7.45	8.60±6.60	7.35±5.42	***	***	**
D2% (Gy)	25.07±11.02	14.24±9.43	13.11±9.51	***	***	*
V5 (%)	83.67±28.38	49.75±39.71	43.99±38.93	***	***	**
V10 (%)	68.36±35.95	30.44±38.59	25.66±32.84	***	***	*
V20 (%)	36.27±31.19	10.77±23.47	5.69±14.18	***	***	*
V30 (%)	6.66±15.42	0.76±2.89	1.00±4.07	**	**	N.S.
Left lung						
Mean (Gy)	5.90±2.24	5.65±1.58	5.62±1.05	N.S.	N.S.	N.S.
D2% (Gy)	37.46±5.31	39.12±2.67	38.34±2.93	N.S.	N.S.	N.S.
V5 (%)	22.84±7.01	23.53±4.48	22.82±3.79	N.S.	N.S.	N.S.
V10 (%)	15.95±6.21	15.40±3.67	14.49±3.21	N.S.	N.S.	*
V20 (%)	11.12±5.55	10.67±3.39	9.83±3.01	N.S.	N.S.	*
V30 (%)	6.52±4.59	6.6±2.90	5.97±2.60	N.S.	N.S.	N.S.

Note: * <0.05, ** <0.01,*** <0.001, N.S.: not significant

**Fig. 2:**
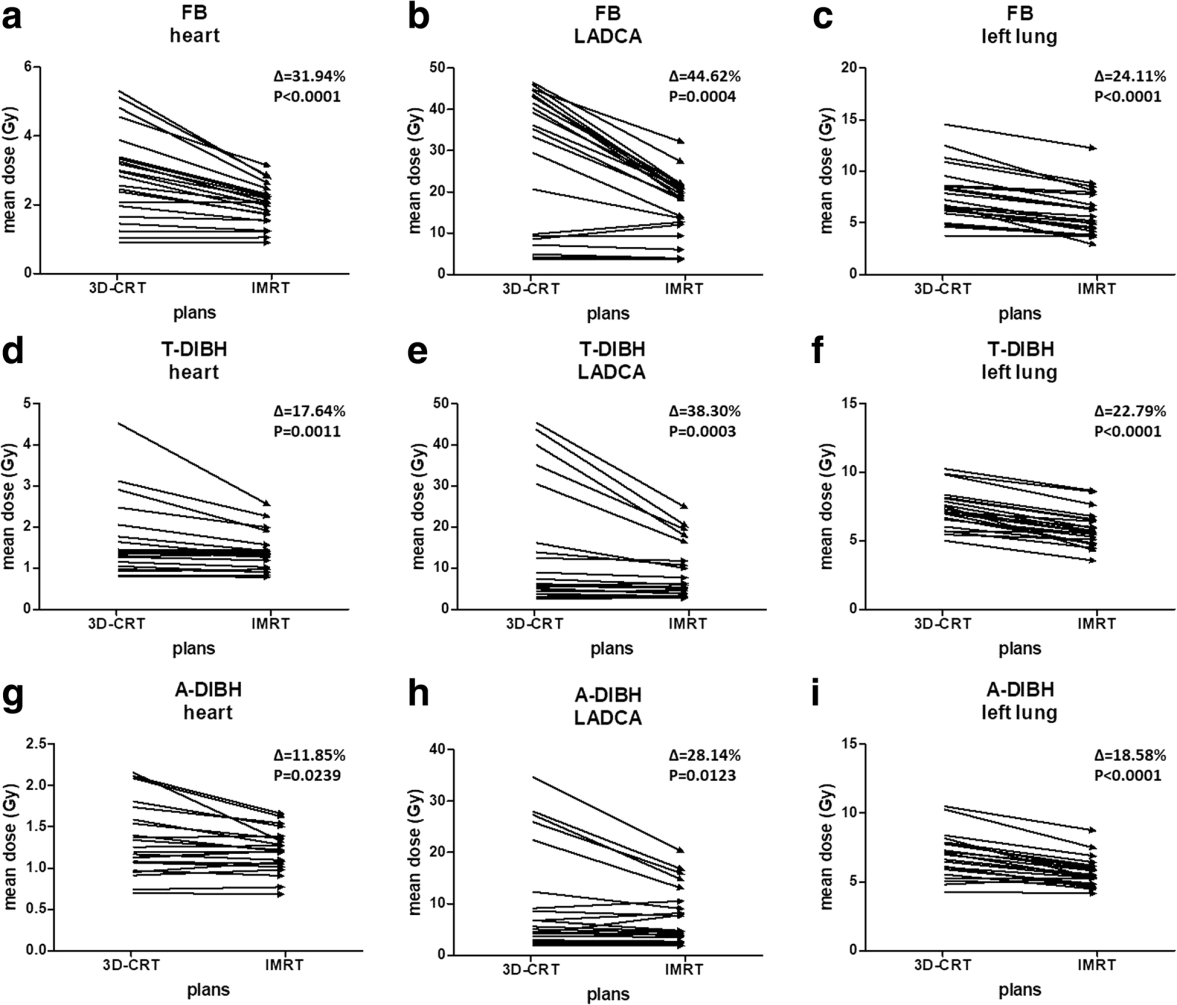
Mean doses to the heart (**a, d, g**), LADCA (**b, e, h**) and left lung (**c, f, i**) in Gy following 3D-CRT and IMRT plans during FB (upper row), T-DIBH (middle row) and A-DIBH (lower row). ∆: mean dose difference between 3D-CRT and IMRT plans

The average doses to the heart, LADCA and left lung in Gy following 3D-CRT and IMRT planning with the 3 different breathing maneuvers are shown in Fig. 2. In the FB, T-DIBH and A-DIBH modes, IMRT resulted in a lower mean dose to the heart, LADCA and left lung. Moreover, IMRT plans with A-DIBH achieved the lowest mean doses to the heart, LADCA and left lung.

## Discussion

Many DIBH techniques have been proposed for use during radiation for treatment of left-sided breast cancer to minimize the cardiac dose, including involuntary breath hold with active breathing control and voluntary breathing using various devices. In 2001, Sixel et al. [9] first reported that a DIBH technique using an active breathing coordinator (ABC) device had the potential to significantly decrease the irradiated cardiac volume during left-sided breast RT. Remouchamps et al. [22] then reported an initial result of a 3.6% reduction of the heart V30 using a moderate DIBH technique with an ABC device. In 2005, Korreman et al. [23] first used an RPM system to evaluate various respiratory maneuvers and demonstrated the dosimetric benefits of FB-gated breast cancer RT. And more recently, Schönecker et al. reported that the Catalyst™/Sentinel™ system (C-RAD) enabled a fast and reliable application and surveillance of DIBH in daily clinical routine and showed that DIBH could significantly reduce high dose areas and mean doses to the heart [24]. Because of its convenience and low cost, voluntary DIBH with RPM has been widely used in the clinic.

However, in both voluntary and involuntary DIBH, positional variation can occur among patients due to the different DIBH maneuvers including T-DIBH and A-DIBH. Before practicing DIBH; in our study, when patients were asked to perform DIBH, the majority of patients (16/22, 72.7%) tended to perform T-DIBH, and the others (6/22, 27.3%) tended to perform A-DIBH. In other words, T-DIBH is the first choice for most patients when performing DIBH. After practice, all patients were able to perform T-DIBH and A-DIBH well at will.

Similar to previous studies, we observed a significantly smaller heart volume during DIBH compared with FB [11]. This reduced heart volume was due to the increased intrathoracic pressure induced by inflation of the lung and might have contributed to the reduced doses to the heart and LADCA. In addition, in our study, the heart volume was not significantly different between T-DIBH and A-DIBH.

In the present study, we focused on comparing three breathing maneuvers, namely, FB, T-DIBH and A-DIBH, in terms of the dosimetric distributions of 3D-CRT and IMRT plans for the first time. For dosimetric comparison, it is important that the target coverage is as equal as possible. We obtained similar target coverage in the FB_3D-CRT, TDIBH_3D-CRT, ADIBH_3D-CRT, FB-IMRT, TDIBH-IMRT, and ADIBH-IMRT plans. Additionally, no significant differences among FB_3D-CRT, TDIBH_3D-CRT, and ADIBH_3D-CRT were observed for the Dmean, D2%, or V105% of the PTV or for the HI. Meanwhile, no significant differences among FB_IMRT, TDIBH_IMRT, and ADIBH_IMRT were observed for the Dmean, D2%, or V105% of the PTV or for the HI.

No Chinese patient data have been reported previously. Therefore, our study is the first to show data from Chinese patients. Our dosimetric data demonstrated the following: 1. The DIBH technique (regardless of whether T-DIBH or A-DIBH is performed) was able to reduce the heart dose and LADCA dose. 2. A-DIBH further reduced the radiation doses to the heart and LADCA compared with T-DIBH. 3. IMRT plans have greater benefits for reducing radiation doses to the heart and LADCA than 3D-CRT plans. For 3D-CRT plans, in our study, the mean heart dose was reduced from 1.67 Gy for T-DIBH to 1.34 Gy for A-DIBH, and the mean LADCA dose was lowered from 13.94 Gy for T-DIBH to 10.22 Gy for A-DIBH. As reported in previous studies in which plans were generated with 3D-CRT techniques [11, 15–17, 25–28], the mean heart dose range was 1.14–6.2Gy in FB plans and 0.84–3.1 Gy in DIBH plans, and the mean LADCA dose range was 6.12–26.26 Gy in FB plans and 1.86–16.01 Gy in DIBH plans. For IMRT plans, in our study, the mean heart dose was reduced from 1.37 Gy for T-DIBH to 1.18 Gy for A-DIBH, and the mean LADCA dose was reduced from 8.6 Gy for T-DIBH to 7.35 Gy for A-DIBH. As reported in previous studies, in which plans were generated with IMRT techniques [13, 14, 16, 18, 25, 29, 30], the mean heart dose range was 1.9–6.9 Gy in FB plans and 0.6–3.9 Gy in DIBH plans, and the mean LADCA dose range was 11.4–31.7 Gy in FB plans and 5.5–21.9Gy in DIBH plans.

Moreover, our dosimetric data demonstrated the following: 1. When selecting a 3D-CRT technique, DIBH (regardless of whether T-DIBH or A-DIBH is performed) can reduce the dose to the ipsilateral lung compared with FB. 2. When selecting the IMRT technique, DIBH (regardless of whether T-DIBH or A-DIBH is performed) cannot further reduce the mean dose to the ipsilateral lung compared with FB. 3. IMRT plans can further reduce the mean dose to the ipsilateral lung compared with 3D-CRT plans. Most previous studies comparing DIBH with FB have consistently showed reduced doses to the heart and LADCA; however, the lung dose results were inconsistent. Several studies [11, 25, 26, 29] have reported that DIBH can significantly reduce the left lung dose, and the majority of these studies used the 3D-CRT technique to generate plans. Certain other studies [13, 15, 27, 31] reported that the mean left lung dose during DIBH was not significantly different from that during FB, but the majority of these studies used the IMRT technique to generate plans. Therefore, when using the 3D-CRT technique, DIBH could contribute to a lower left lung dose; if using the IMRT technique, the left lung dose is similar in FB or DIBH mode.

Our study has a primary limitation that our study cohort size of 22 patients is modest. In our study, in fact, nearly every patient’s dosimetric data indicated that A-DIBH had better outcomes than T-DIBH; therefore, we stopped recruiting additional patients.

## Conclusions

In summary, our study focused on different breathing maneuvers for DIBH during RT for left-sided breast cancer and found the following: 1. A-DIBH_3D-CRT plans had a greater effect than T-DIBH_3D-CRT plans on the heart, LADCA, and left lung doses. 2. A-DIBH_IMRT plans had a greater effect than T-DIBH_IMRT plans on the heart and LADCA doses, but had no positive effect compared with T-DIBH_IMRT plans on the left lung dose; 3. A-DIBH_IMRT plans had the lowest doses to the heart, LADCA and left lung doses.

Clinically, therefore, these findings indicate the following: 1. It is not sufficient to simply ask patients to practice DIBH before simulation; rather, it is important to train patients to practice A-DIBH before simulation as well as to perform A-DIBH during both simulation and RT. 2. If the equipment conditions and technology are feasible, the A-DIBH technique combined with the IMRT planning technique is strongly recommended for RT of left breast cancer patients.
